# Identification of gene-based responses in human blood cells exposed to alpha particle radiation

**DOI:** 10.1186/1755-8794-7-43

**Published:** 2014-07-12

**Authors:** Vinita Chauhan, Matthew Howland, Ruth Wilkins

**Affiliations:** 1 Consumer and Clinical Radiation Protection Bureau, Healthy Environment and Consumer Safety Branch, Health Canada, 775 Brookfield Road, PL 6303B, Ottawa, ON K1A 1C1, Canada

**Keywords:** α-particles, Gene expression, Leukocytes

## Abstract

**Background:**

The threat of a terrorist-precipitated nuclear event places humans at danger for radiological exposures. Isotopes which emit alpha (α)-particle radiation pose the highest risk. Currently, gene expression signatures are being developed for radiation biodosimetry and triage with respect to ionizing photon radiation. This study was designed to determine if similar gene expression profiles are obtained after exposures involving α-particles.

**Methods:**

Peripheral blood mononuclear cells (PBMCs) were used to identify sensitive and robust gene-based biomarkers of α-particle radiation exposure. Cells were isolated from healthy individuals and were irradiated at doses ranging from 0-1.5 Gy. Microarray technology was employed to identify transcripts that were differentially expressed relative to unirradiated cells 24 hours post-exposure. Statistical analysis identified modulated genes at each of the individual doses.

**Results:**

Twenty-nine genes were common to all doses with expression levels ranging from 2-10 fold relative to control treatment group. This subset of genes was further assessed in independent complete white blood cell (WBC) populations exposed to either α-particles or X-rays using quantitative real-time PCR. This 29 gene panel was responsive in the α-particle exposed WBCs and was shown to exhibit differential fold-changes compared to X-irradiated cells, though no α-particle specific transcripts were identified.

**Conclusion:**

Current gene panels for photon radiation may also be applicable for use in α-particle radiation biodosimetry.

## Background

Nuclear terrorism is a global concern with illicit trafficking events involving nuclear material on the rise [1]. Between 1993 and 2011, the International Atomic Energy Association (IAEA) documented 2164 nuclear material incidents or malicious acts and 588 involved the theft or loss of nuclear or radioactive materials. A further 18 of these involved plutonium or highly enriched uranium. Such events highlight the potential for radioactive material to fall into the wrong hands and potentially being used for the fabrication of a radiological dispersal device (RDD) [2]. Los Alamos National Laboratory has conducted a thorough review of RDD source material and has postulated that four of the nine isotopes most likely to be employed are α-particle emitters, primarily due to their minimal shielding requirements and ease of concealment [3]. There is also the concern of long-term contamination due to these isotopes’ long half-lives and the severe biological damage that can occur from a minimal dose of exposure.

In recent years, much work has gone towards developing strategies for radiation biodosimetry with a specific focus on photon radiation [4-8]. However, the strategies currently employed for photon radiation may not provide adequate dose estimates for α-particle exposures. Unlike photon radiation, α-particles travel a short distance (40-70 μm) and create very dense ionizing tracks as they traverse a medium. They typically cause an energy deposition of 160 keV•μm^-1^ for 2.5 MeV α-particles in comparison to 2.0 keV•μm^-1^ for low linear energy transfer (LET) X-rays [9,10]. Therefore, α-particles produce more significant biological effects when compared to equal absorbed doses from low-LET photon radiation, which are more sparsely ionizing [11-13]. This difference in ionization density may provide a means of distinguishing radiation type based on the magnitude of the biological response.

Development of biomarker-based biodosimetry has been put forth as one of the key priority development areas for nuclear threat countermeasures [14], and microarray data/gene based profiling has served as timely and minimally invasive means to address this priority area [15]. There have been several studies examining the gene expression profiles of human cells using functional genomics platforms for photon radiation [16-18]. However, the availability of similar gene tools for high LET radiation types, such as α-particles, remains limited. To date, the majority of α-particle transcriptional studies have been performed *in vitro* using transformed or normal cell types [19-21]. There has also been a selected few studies that have profiled genomic changes and compared the responses following exposure of cells to different radiation types [22,23]. Microarray studies in our own lab using epidermal keratinocytes exposed to both α-particle and X-ray radiation have also shown transcriptional differences between these radiation types [23].

In the present study, genomic strategies were employed to identify biomarkers of α-particle radiation exposure. Circulating peripheral blood mononuclear cells (PBMCs) were isolated from normal, healthy volunteers and exposed to α-particle radiation. Twenty-four hours post-exposure, the expression of transcripts was assessed using Illumina bead array technology and these responses were compared to non-irradiated controls. Dose-responsive genes were then further assessed in independent white blood cell (WBC) populations exposed to either α-particles or X-rays.

## Methods

### Blood draws

**Figure 1:**
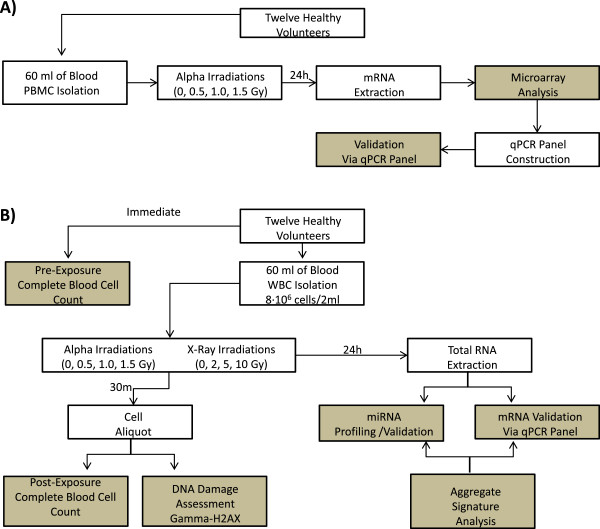
Schematic representation of the experimental process, assays and endpoints (those yielding data are shaded) for the A) peripheral blood mononuclear cell and B) the white blood cell populations.

All procedures were approved by Health Canada’s Research Ethics Committee and a flow chart delineating the experimental sequence is outlined in Figure 1. Briefly, peripheral blood from healthy, non-smoking volunteers was drawn via periphery venipuncture with informed consent from all subjects into either 5 × 10 ml EDTA (for gene analysis) or 2 × 4 ml lithium heparin (for plasma analysis) vacutainer tubes (Becton Dickinson and Company, Franklin Lakes, NJ). A total of 6 male and 6 female donors participated. Before any further processing, a 100 μl whole blood sample was used for a complete blood count (CBC) via automatic haemocytometer (Beckman Coulter, Mississauga, ON).

### Peripheral blood mononuclear cell isolation

PBMCs were isolated from whole blood for an initial global screening of gene transcripts using microarray technology. A similar isolation procedure was employed as described by Boyum [24]. Briefly, 15 ml of Histopaque-1077 sucrose gradient (Sigma-Aldrich, MO, USA) was pipetted into the upper chamber of an Accuspin Tube (Sigma-Aldrich). The tube was centrifuged (800 × g) for 30 sec to ensure that the Histopaque was below the frit layer. Freshly drawn whole blood was pipetted into the upper chamber of tube. The tube was then centrifuged 800 × g for 15 minutes. The band of mononuclear cells was transferred to an alternate centrifuge tube and washed with 10 ml of isotonic phosphate buffered saline (PBS) three times. Pelleted cells were then resuspended in RPMI-1640 media supplemented with 10% fetal bovine serum (FBS), 2 mM L-glutamine and 100 U penicillin & 100 μg streptomycin/ml (Sigma-Aldrich).

### Total white blood cell isolation

Following initial experiments on microarray analysis of PBMCs, further studies using qPCR were conducted on complete WBC populations. WBCs were isolated from whole blood using Histopaque-1119 (Sigma-Aldrich). Twenty-five milliliters of whole blood was gently poured over 15 ml of Histopaque-1119 and spun at 1000 × g for 10 minutes. This resulted in erythrocyte sedimentation below the Histopaque gradient and a total white blood cell population above the gradient. This volume (~15 ml) was then transferred to a new 50 ml tube and diluted 1:2 with phosphate buffered saline (PBS). The resulting WBC pellet was then washed twice more with 10 ml PBS and resuspended in RPMI-1640 media supplemented with 10% FBS, 2 mM L-glutamine and 100U penicillin & 100 μg streptomycin/ml.

### PBMCs and WBC irradiations

Either Isolated PBMCs or WBCs were seeded at total cell density of 8-10 × 10^6^ cells in 2ml of media and were cultured on thin Mylar based plastic dishes (MD) (Chemplex Industries, Palm City, FL, USA), which allowed for penetration of α-particles. Cells were allowed to settle for 10 minutes before performing the irradiations. Irradiations were performed at doses of 0 (control), 0.5, 1.0 or 1.5 Gy using Americium (^241^Am) electroplated discs with an activity level of 66.0 kBq ± 3% (dose rate of 0.98 ± 0.01 Gy/h, LET of 127.4 ± 0.4 keV/μm). The absorbed dose of α-particle radiation to which the cells were exposed was calculated using the GEANT4 v.9.1 Monte Carlo tool-kit [25]. Cells destined for X-ray radiation at doses of 0 (control) 2, 5 or 10 Gy were exposed using the X-RAD 320 X-ray irradiation system (Precision X-ray, Inc., North Branford, CT, USA) at a higher dose rate of 0.98 ± 0.05 Gy/min. Exposures were performed in duplicate and pooled. Twenty-four hours following irradiation, a 50 μl aliquot of cells was assessed for cellular viability using the Trypan Blue viability assay (Bio-Rad, Hercules, CA), and a 100 μl aliquot was used for a CBC via automatic haemocytometer. The remainder of the cells were spun down and used for RNA extraction.

### H2AX phosphorylation assay

H2AX phosphorylation was assessed using flow cytometry following a modified protocol by MacPhail et al. [26]. Thirty minutes after exposure, WBC suspensions (5 × 10^5^ cells per sample) were fixed with 10% formaldehyde (Fisher Scientific, USA) and incubated for 10 min at room temperature. The cells were then washed and re-suspended in 1 ml cold (−40°C), 70% methanol (Fisher Scientific) in 1x PBS and stored at -40°C overnight or up to two weeks. One ml of cold TBS (tris-phosphate buffered saline, 0.0154 M Trizma Hydrochloride (Sigma–Aldrich Canada), 0.5 M NaCl (Fisher Scientific), pH 7.4) was then added to each sample, mixed well, centrifuged (8 min, 400 × g, 4°C) and re-suspended in 1 ml of cold TST (TBS serum triton, 96% TBS, 4% FBS (Sigma–Aldrich), 0.1% Triton X-100 (Sigma–Aldrich)). The samples were incubated on ice for 10 min, centrifuged (5 min, 400 × g, 4°C) and re-suspended in 200 μl of anti-γ-H2AX-fluorescein isothiocyanate (FITC) antibody (Millipore, USA) diluted 1:500 in TST. After 2 h incubation on ice in the dark, 1 mL of TBS with 2% FBS was added. The samples were then centrifuged (5 min, 400 × g, 4°C), re-suspended in 250 μl TBS with 2% FBS. Immediately prior to analysis by flow cytometry, 2 μl of 1 mg/mL propidium iodide (PI) was added to each sample. For flow cytometry analysis, data acquisition was set to analyze 2 × 10^4^ cells from the whole cell population as identified by a forward scatter (FSC) vs. side scatter (SSC) dot plot. All debris under the FSC and SSC threshold were excluded from the analysis. The γ-H2AX response was measured by assessing the increased level of intracellular fluorescence characterized in the cells, as determined by the geometric mean of the intensity peak of the anti-γ-H2AX-FITC (channel number) of the γ-H2AX positive cells. All samples were analyzed on a BD FACSCalibur flow cytometer (BD Biosciences, San Jose, CA, USA).

### RNA extractions

Twenty-four hours post-radiation exposure or negative control conditions, RNA extractions were performed on either PBMCs or WBCs. In the case of PBMCs, the cells were resuspended in 350 μl of Buffer RLT containing 1% β-Mercaptoethanol (Qiagen’s RNeasy Mini kit; Qiagen Inc, Mississauga, ON) then frozen at -80°C until processed. Frozen lysates were thawed on ice and mixed well by pipetting. The lysate was transferred directly onto a QIAshredder spin column (Qiagen Inc), placed in a 2 ml collection tube and centrifuged for 2 min at ~12,000 g. A volume of 350 μL of 70% ethanol was added. Total RNA was then extracted using the RNeasy Mini kit according to the manufacturer’s instructions (Qiagen Inc), with the addition of Qiagen’s On-Column RNase-free DNase (Qiagen Inc) to eliminate any remaining DNA contamination. In the case of the WBCs, RNA extractions were performed using QIAzol reagent (Qiagen) and following manufacturer’s instructions. Briefly, 700 μl of QIAzol reagent was added to the cell pellet and then homogenized via up-and-down pipetting of the mixture 50 times. After room temperature incubation for 15 minutes, 140 μl of chloroform was added for phase separation. The aqueous layer containing RNA was then removed and precipitated with 100% ethanol. Total RNA was then isolated using the miRNeasy column purification kit. All total RNA sample concentrations and RNA quality were determined using both an Agilent 2100 Bioanalyzer and RNA Nanochips (Agilent Technologies Canada Inc., Mississauga, ON) and spectrophotometrically (OD ratio of A260:A280) using a Nanodrop (Fisher Scientific, Ottawa, ON). All extracted PBMC RNA samples were determined to be of good quality (RNA Integrity Number ≥ 8.0) with minimal degradation and stored at -80°C until further analysis. WBC RNA from α-particle exposed samples was determined to be of good quality (RNA Integrity Number ≥ 9.2) with three samples being excluded from sample analysis due to insufficient RNA yield.

### Genomic profiling

An input of 200 ng of PBMC mRNA was used for whole genome analysis following the Illumina(r) Whole Genome Expression Profiling Assay Guide (11317302 Rev. A). Samples were hybridized on Illumina human-12 v2 RNA BeadChips. BeadChips were imaged and quantified with the Illumina iScan scanner and data was processed with Illumina GenomeStudio v2010.2.8.11.

### MiRNA profiling

An input of 200 ng of WBC miRNA expression was profiled using the nCounter system (NanoString Technologies, Seattle, WA) which profiles the expression levels of miRNAs. This was performed using the human miRNA expression assay (version 2) according to manufacturer’s instructions and read using the nCounter digital analyser.

### Quantitative real time-polymerase chain reaction (qPCR) validation

Selected genes deemed statistically significant (as described below in the Statistical analysis section) by microarray analysis or nCounter system were further assessed by qPCR. Total RNA (400 ng mRNA and 200 ng miRNA) isolated from cells were reverse transcribed into complementary DNA (cDNA) using the RT2 First Strand Kit (Qiagen) or miScript Kit respectively. Gene profiling was performed according to the manufacturer’s instructions using custom RT2-profiler PCR arrays (Qiagen). Reactions were prepared in 96-well plates and performed using a spectrofluorometric thermal cycler (Biorad iCycler; Hercules, CA). The relative expression of each gene was determined by using the comparative threshold (Ct) method [27].

### Customized gene array panel

A total of 84 unique identifiers were used for the development of a customized 384-well format gene array panel (Table 1). This panel was comprised of genes that were shown by microarray technology to be dose-responsive and also expressed at 1.0 and 1.5 Gy following exposure of PBMC to α-particle radiation. This panel also included a negative control gene (*GNG7*), housekeeping genes (*ACTB*, *GAPDH, GUSB, B2M*) and selected few genes derived from the work of Paul and Amundson [16] which were not identified in this study as statistically significant by microarray analysis (Table 1). SABiosciences (Qiagen) designed the primers and provided a 384 well-format platform that was compatible for use on the LightCycler 480 real-time PCR system (Roche, Mississauga, ON). A high-throughput PCR platform, comprising the Caliper Zephyr Compact Liquid Handling Station, the Caliper Twister II plate handler (PerkinElmer, Woodbridge, ON) and the Lightcycler 480 was employed with custom protocols developed in the Inhalation Toxicology Laboratory of Health Canada. This system allowed for the screening of 144 samples in a one-week time-span.

**Table 1:** Summary of transcripts on customized gene array

**Alpha (All dose)**		**Alpha (Med/High doses)**		**X-ray**		**Controls**	
**Gene ID**	**RefSeq #**	**Gene ID**	**RefSeq #**	**Gene ID**	**RefSeq #**	**Gene ID**	**RefSeq #**
*Acta2*	Nm_001613	*Ankra2*	Nm_023039	*Anxa4*	Nm_001153	*Gng7*	Nm_000546
*Aen*	Nm_022767	*Arhgef3*	Nm_019555	*Ei24*	Nm_004879	*Gapdh*	Nm_004048
*Apobec3h*	Nm_181773	*Bbc3*	Nm_014417	*Il21r*	Nm_021798	*Gusb*	Nm_002046
*Ascc3*	Nm_022091	*Btg3*	Nm_006806	*Ly9*	Nm_002348	*B2m*	Nm_000181
*Astn2*	Nm_198187	*Ccdc90b*	Nm_021825	*Mettl7a*	Nm_014033	*Hgdc*	Sa_00105
*Bax*	Nm_004324	*Cd70*	Nm_001252	*Myc*	Nm_002467	*Rtc*	Sa_00103
*Ccng1*	Nm_004060	*Cdkn1a*	Nm_000389	*Plk2*	Nm_006622	*Ppc*	Sa_00104
*Cmbl*	Nm_138809	*Dcp1b*	Nm_152640	*Plk3*	Nm_004073		
*Ddb2*	Nm_000107	*Dram1*	Nm_018370	*Ptp4a1*	Nm_003463		
*Fas*	Nm_000043	*E2f7*	Nm_203394	*Rasgrp2*	Nm_001098670		
*Fbxo22*	Nm_012170	*Eda2r*	Nm_021783	*Slc4a11*	Nm_032034		
*Gadd45a*	Nm_001924	*Fam127b*	Nm_001078172	*Tcf3*	Nm_003200		
*GlS2*	Nm_013267	*Fam20b*	Nm_014864	*Urod*	Nm_000374		
*Ier5*	Nm_016545	*Fdxr*	Nm_004110	*Vwce*	Nm_152718		
*Mamdc4*	Nm_206920	*Fhl2*	Nm_001450	*Tp53*	Nm_052847		
*Map4k4*	Nm_004834	*Gdf15*	Nm_004864	*Lig1*	Nm_000234		
*Pcna*	Nm_182649	*Gss*	Nm_000178				
*Phlda3*	Nm_012396	*Hist1h4b*	Nm_003544				
*Phpt1*	Nm_014172	*Igfbp4*	Nm_001552				
*Ppm1d*	Nm_003620	*Iscu*	Nm_014301				
*Rps27l*	Nm_015920	*Isg20*	Nm_002201				
*Sesn1*	Nm_014454	*Lamc3*	Nm_006059				
*Tmem30a*	Nm_018247	*Mdm2*	Nm_002392				
*Tnfrsf10b*	Nm_003842	*Nudt15*	Nm_018283				
*Tnfrsf10d*	Nm_003840	*Pcnxl2*	Nm_014801				
*Tnfsf4*	Nm_003326	*Polh*	Nm_006502				
*Tp53inp1*	Nm_033285	*Prkab1*	Nm_006253				
*Triap1*	Nm_016399	*Pvt1*	Nr_003367				
*Xpc*	Nm_004628	*Retsat*	Nm_017750				
		*SAC3D1*	NM_013299				
		*SLC7A6*	NM_003983				
		*TMPRSS7*	NM_001042575				
		*TNFSF8*	NM_001244				
		*TOB1*	NM_005749				
		*TP53AP1*	NR_015381				
		*TRIM22*	NM_006074				
		*TRIM32*	NM_012210				
		*ZNF337*	NM_015655				
		*ZNF79*	NM_007135				

The array includes genes differentially expressed at all doses (0.5 - 1.5 Gy) and those only at the medium (1.0 Gy) and high (1.5 Gy) dose derived from microarray analysis of PBMCs exposed to α-particle radiation. X-ray column represents genes that were not differentially expressed in this study but shown to be X-ray responsive, primarily from the work of Paul and Amunduson. *GNG7* is a negative control. *GAPDH, GUSB, B2M* are housekeeping genes. Human Genomic DNA Control (HGDC), Reverse Transcription Control (RTC) and Positive Primer Control (PPC) are internal controls specific to the SABiosystems experimental system.

### Statistical analysis

Microarrays statistical analysis was performed as follows. Data background correction was done within GenomeStudio (Illumina), and then exported to the lumi R package. Data was then normalized via quantile method, rendering the distribution of probe intensities of each array in a set of arrays equivalent. Normalized data was then log2 transformed for statistical comparisons. Linear models for microarray data (LIMMA) was employed to identify differentially expressed gene signatures between the different exposure conditions for the PBMC microarray, nCounter miRNA and the WBC qPCR datasets [28,29]. In brief, this method involves fitting a linear model for each gene in the data and moderating the standard error via an empirical Bayes method. This is used to estimate the moderated t-statistics/F-statistics for each gene, shrinking the standard error towards a common value. This test is similar to an ANOVA for each gene with the exception that standard deviations are moderated across genes, allowing more stable inference for each gene. Moderated standard deviations are a compromise between individual genewise standard deviations and overall pooled standard deviations. Multiple comparison false discovery rate (FDR) was evaluated using the Benjamini-Hochberg (BH) method [30].

PBMC qPCR data was analysed for statistical significance without multiple correction comparison using gene-wise ANOVA as there was a-priori reason for gene analysis. Hierarchical clustering was performed using the WBC qPCR data using dChip (http://www.hsph.harvard.edu/cli/complab/dchip). This software was used to cluster the different exposure conditions by gene signature and group genes by similarity of expression patterns. The distance between genes is measured as 1- r (Pearson correlation coefficient).

## Results

### DNA damage

**Figure 2:**
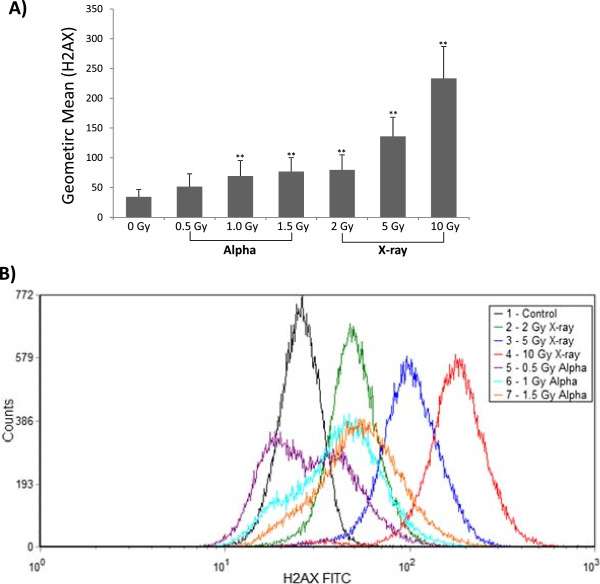
**Isolated white blood cells were used to assess H2AX phosphorylation expression after exposure to α-particle or X-ray radiation. A)** Geometric mean of signal intensity (indicative of γ-H2AX expression) for each of the doses and radiation types. Data is presented as means ± SD with n = 12 biological replicates. **represents p < 0.01 **B)** Representative flow cytometric histogram overlay of γ-H2AX expression in WBC cells at various doses of α-particle and X-ray exposure measured 30 min post-exposure.

To ensure that the WBC suspensions were undergoing irradiation and sustaining DNA damage, a biological assay indicative of DNA damage, the phosphorylation of H2AX, was employed. Thirty minutes post-exposure, cells were assessed for the expression of γ-H2AX, a marker indicative of DNA double strand breaks. A dose-dependent increase in the γ-H2AX signal was observed following exposure to α-particle radiation as seen by a plot of the geometric mean of this signal as a function of dose and the pronounced shift in the curve (Figure 2). Statistically significant responses were obtained at the medium (1.0 Gy) and high (1.5 Gy) doses tested (p < 0.01) relative to the non-irradiated control treatment group. A bi-modal shaped curve was observed at the lowest dose of α-particle radiation which with increasing doses transitioned to a mono-modal curve. At the highest dose (1.5 Gy) an approximate 3-fold increase in γ-H2AX signal was observed relative to the control sample. As a positive control, isolated leukocytes were irradiated with X-rays at a high dose rate (1 Gy/min) and a dose range of 2-10 Gy. A plot of this response indicated X-rays to induce more double strand breaks as seen by the marked increase in γ-H2AX signal with dose of radiation relative to α-particle treated cells.

### Genomic profiling

**Figure 3:**
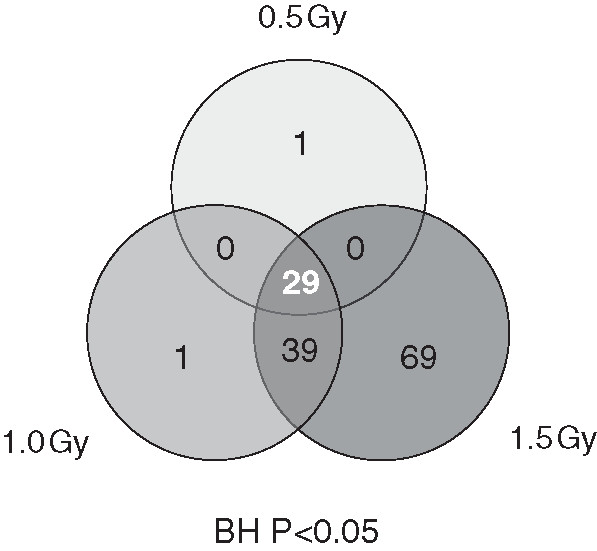
**Venn diagram showing overlap patterns of genes which were show to be significantly modulated via microarray in peripheral blood mononuclear cells after various doses of α-particle radiation.** Based on an n = 12 human donors.

**Figure 4:**
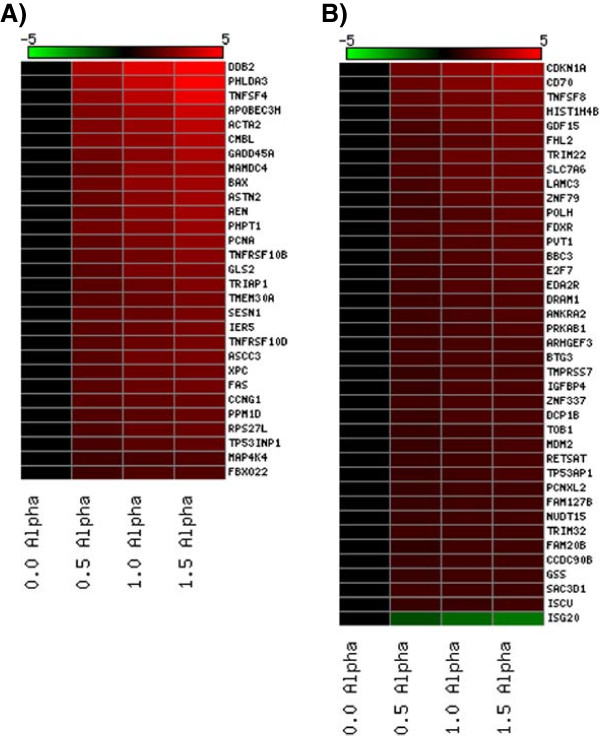
**Heat map depicting PBMC fold change microarray gene expression values from those shown to be statistically significant at all A) three doses (0.5, 1.0, 1.5 Gy) and B) the medium (1.0 Gy) and high (1.5 Gy) dose.** Red colouring signifies up-regulation and green colouring signifies down-regulation based on an n = 12 biological replicates.

Genomic profiling was performed on RNA extracted from isolated PBMCs 24 hr post-exposure. In order to identify biomarkers, statistical stringency was prioritized to mine for reliable genes using a Benjamini Hochberg (BH) false discovery rate (FDR) correction. All differentially expressed genes were filtered on flagged spots and a BH FDR corrected p-value <0.05. A numeric summary of the gene responses at each of the doses is provided in Table 2. Overall, there was a pronounced induction of transcriptional response, with the majority of genes being up-regulated in the presence of the radiation insult. Escalating doses induced an increasing number of transcripts with 30, 69 and 137 genes differentially modified at 0.5, 1.0 and 1.5 Gy respectively. A Venn diagram was constructed to provide a quantitative representation of the similarities and differences in expression profiles at each of the doses (Figure 3). Twenty-nine genes were shown to be differentially expressed at all three doses with expression levels ranging from 2-10 fold. Thirty-nine genes were common between differentially expressed gene sets at both the medium (1.0 Gy) and high (1.5 Gy) dose. The range in expression levels of these 68 genes is summarized as a heat map which delineates the genes by degree of fold change (Figure 4).

**Table 2:** Numeric summary of differentially expressed transcripts that were obtained from exposure of isolated PBMC to α-particle radiation and categorized by dose

	**0.5 Gy**	**1.0 Gy**	**1.5 Gy**
Number of transcripts	30	69	137
Common amongst doses *(%)*	29 (97)	29 *(42)*	29 *(21)*
Exclusive *(%)*	1 (3)	1 *(1)*	69 *(50)*
Up regulated *(%)*	29 (97)	68 *(99)*	120 *(88)*
Common amongst doses *(%)*	29 (100)	29 *(42)*	29 *(23)*
Exclusive *(%)*	0 (0)	1 *(100)*	53 *(39)*
Down regulated *(%)*	1 (3)	1 *(1)*	17 *(12)*
Common amongst all doses *(%)*	0 (0)	0 *(0)*	0 *(0)*
Exclusive *(%)*	1 (100)	0 (0*)*	16 *(12)*

Bracketed numbers indicate the percentage of transcripts that were expressed in each of the categories from the total number of genes responding at each dose.

### QPCR validation

All 68 genes observed to be differentially expressed were further validated using qPCR. A comparison of the responses using the two technologies showed a similar trend in differential expression levels. As shown in Table 3, all genes that exhibited a significant response across the 3 doses using microarray analysis were also observed to exhibit a similar trend using qPCR. However, approximately 20% of the total validated targets were shown to be non-significant using qPCR. This is not unexpected as in comparison to microarrays, qPCR may report different statistical assignments [31]. In contrast, there were also a sub-set of 9 genes which showed statistical significance at the 0.5 Gy dose via qPCR but not using microarray analysis.

**Table 3:** Validation of PBMC gene responses identified by microarray via qPCR

**Radiation type**	**0.5 Gy alpha**				**1.0 Gy alpha**				**1.5 Gy alpha**			
**Technology**	**qPCR**		**MA**		**qPCR**		**MA**		**qPCR**		**MA**	
**Symbol**	**FC**	**PV**	**FC**	**PV**	**FC**	**PV**	**FC**	**PV**	**FC**	**PV**	**FC**	**PV**
**All doses**												
*TRIAP1*	2.15	0.00	1.81	0.00	3.32	0.00	2.14	0.00	3.78	0.00	2.57	0.00
*GADD45A*	2.94	0.00	2.27	0.01	4.43	0.00	2.77	0.00	4.70	0.00	3.38	0.00
*RPS27L*	2.01	0.00	1.67	0.01	2.59	0.00	1.90	0.00	3.57	0.00	2.04	0.00
*MAP4K4*	1.51	0.01	1.40	0.05	1.79	0.00	1.54	0.00	1.90	0.00	1.69	0.00
*TNFRSF10D*	2.15	0.01	1.81	0.01	2.94	0.00	2.08	0.00	3.08	0.00	2.39	0.00
*ASTN2*	6.18	0.01	2.07	0.01	8.02	0.00	2.67	0.00	8.98	0.00	3.15	0.00
*TNFRSF10B*	2.28	0.01	2.02	0.01	3.06	0.00	2.32	0.00	3.13	0.00	2.72	0.00
*TMEM30A*	1.92	0.01	1.72	0.01	2.67	0.00	2.13	0.00	2.76	0.00	2.45	0.00
*TP53INP1*	1.56	0.01	1.53	0.04	1.83	0.00	1.81	0.00	1.88	0.00	2.02	0.00
*BAX*	2.42	0.01	2.31	0.00	3.17	0.00	2.88	0.00	3.89	0.00	3.23	0.00
*FAS*	1.89	0.01	1.79	0.00	2.61	0.00	2.06	0.00	2.94	0.00	2.23	0.00
*DDB2*	2.96	0.01	3.56	0.00	5.15	0.00	4.41	0.00	5.26	0.00	5.49	0.00
*AEN*	4.24	0.01	2.16	0.01	7.09	0.00	2.80	0.00	7.46	0.00	3.14	0.00
*GLS2*	1.94	0.01	1.87	0.01	2.30	0.00	2.37	0.00	2.46	0.00	2.62	0.00
*CMBL*	3.99	0.01	2.52	0.01	6.79	0.00	2.90	0.00	7.41	0.00	3.57	0.00
*PHPT1*	2.28	0.02	2.23	0.00	3.50	0.00	2.57	0.00	4.17	0.00	3.03	0.00
*PCNA*	2.49	0.02	2.02	0.00	3.64	0.00	2.44	0.00	4.09	0.00	2.88	0.00
*ASCC3*	2.14	0.03	1.84	0.00	3.18	0.00	2.22	0.00	3.28	0.00	2.37	0.00
*PPM1D*	1.58	0.05	1.63	0.01	2.14	0.00	1.81	0.00	2.17	0.00	2.11	0.00
*XPC*	1.62	0.05	1.81	0.01	2.20	0.00	2.12	0.00	2.00	0.01	2.31	0.00
*IER5*	2.09	0.05	1.82	0.01	2.71	0.00	2.10	0.00	3.09	0.00	2.41	0.00
*TNFSF4*	2.91	0.06	2.93	0.00	4.98	0.00	3.69	0.00	5.34	0.00	4.67	0.00
*SESN1*	1.40	0.06	1.90	0.00	2.25	0.00	2.14	0.00	1.98	0.01	2.44	0.00
*FBXO22*	1.40	0.08	1.39	0.03	1.97	0.01	1.56	0.00	2.17	0.00	1.65	0.00
*PHLDA3*	9.91	0.09	3.18	0.00	22.89	0.00	3.96	0.00	28.82	0.00	4.89	0.00
*ACTA2*	2.12	0.09	2.54	0.01	2.72	0.01	3.07	0.00	2.74	0.01	3.60	0.00
*CCNG1*	0.91	0.10	1.80	0.00	2.49	0.00	1.97	0.00	2.56	0.00	2.14	0.00
*MAMDC4*	2.54	0.14	2.06	0.04	3.18	0.02	2.82	0.00	3.52	0.01	3.23	0.00
*APOBEC3H*	2.38	0.55	2.52	0.04	3.68	0.08	3.20	0.00	4.49	0.02	3.91	0.00
**Med/High doses**												
*SAC3D1*	1.47	0.01	1.26	0.24	1.74	0.00	1.29	0.04	1.91	0.01	1.33	0.01
*PVT1*	2.02	0.01	1.42	1.00	2.76	0.00	1.68	0.02	3.45	0.00	1.85	0.00
*TP53TAP1*	1.42	0.01	1.20	1.00	1.60	0.00	1.39	0.03	1.92	0.01	1.45	0.01
*TNFSF8*	2.40	0.01	1.92	0.08	4.01	0.00	2.33	0.00	3.80	0.00	2.61	0.00
*TMPRSS7*	4.04	0.01	1.24	1.00	4.85	0.01	1.53	0.00	6.74	0.00	1.52	0.00
*ZNF79*	1.74	0.02	1.39	0.47	2.07	0.00	1.74	0.00	2.34	0.00	1.95	0.00
*SLC7A6*	1.64	0.03	1.62	0.15	1.98	0.01	1.89	0.00	2.22	0.00	2.15	0.00
*CDKN1A*	2.41	0.03	2.30	0.11	3.87	0.00	2.94	0.00	5.05	0.00	3.58	0.00
*MDM2*	2.93	0.04	1.24	0.31	4.44	0.00	1.30	0.02	5.17	0.00	1.45	0.00
*FDXR*	6.46	0.06	1.47	0.85	13.07	0.00	1.76	0.01	14.43	0.00	1.88	0.00
*DCP1B*	1.68	0.06	1.17	1.00	2.00	0.00	1.45	0.01	2.33	0.00	1.51	0.00
*BBC3*	4.10	0.06	1.40	0.66	6.75	0.00	1.65	0.01	7.25	0.00	1.83	0.00
*ISG20*	0.75	0.06	-1.44	1.00	0.46	0.00	-1.92	0.00	0.46	0.00	-2.30	0.00
*FAM20B*	1.49	0.07	1.08	1.00	1.45	0.01	1.25	0.03	1.55	0.01	1.35	0.00
*DRAM1*	1.41	0.07	1.32	1.00	1.70	0.00	1.55	0.03	1.91	0.00	1.66	0.00
*CCDC90B*	2.18	0.07	1.20	0.64	1.54	0.07	1.29	0.02	2.89	0.00	1.35	0.00
*POLH*	1.72	0.08	1.39	0.24	2.37	0.00	1.60	0.00	2.10	0.01	1.92	0.00
*GSS*	1.19	0.08	1.15	1.00	1.34	0.01	1.29	0.03	1.60	0.00	1.34	0.00
*PRKAB1*	1.40	0.08	1.29	0.59	1.62	0.01	1.49	0.00	1.85	0.00	1.60	0.00
*ARHGEF3*	1.37	0.09	1.32	0.59	1.74	0.01	1.51	0.00	1.68	0.02	1.55	0.00
*ZNF337*	1.23	0.11	1.34	0.25	1.53	0.02	1.44	0.01	1.61	0.02	1.52	0.00
*HIST1H4B*	1.34	0.11	1.83	0.75	1.62	0.02	2.21	0.03	1.79	0.01	2.59	0.00
*LAMC3*	2.76	0.12	1.54	1.00	3.63	0.00	1.93	0.02	4.50	0.00	2.13	0.00
*GDF15*	1.84	0.14	1.49	1.00	2.40	0.01	1.98	0.03	2.96	0.05	2.26	0.00
*ISCU*	1.15	0.15	1.18	0.98	1.29	0.01	1.24	0.05	1.47	0.00	1.26	0.01
*TRIM22*	1.28	0.15	1.69	0.25	1.96	0.02	2.06	0.00	1.63	0.02	2.16	0.00
*BTG3*	1.11	0.23	1.30	0.21	1.69	0.00	1.41	0.00	1.70	0.01	1.54	0.00
*NUDT15*	1.22	0.23	1.21	0.24	1.48	0.02	1.31	0.00	1.47	0.03	1.38	0.00
*RETSAT*	1.25	0.27	1.21	1.00	1.16	0.56	1.38	0.03	1.19	0.29	1.45	0.00
*TRIM32*	1.32	0.27	1.25	0.45	1.10	0.15	1.34	0.02	1.58	0.04	1.37	0.00
*FHL2*	1.74	0.30	1.50	0.44	2.47	0.02	1.82	0.00	2.65	0.01	2.20	0.00
*FAM127B*	1.14	0.30	1.21	0.54	1.53	0.01	1.31	0.00	1.58	0.05	1.38	0.00
*PCNXL2*	1.23	0.37	1.19	1.00	1.18	0.52	1.33	0.01	1.34	0.15	1.40	0.00
*E2F7*	2.23	0.37	1.33	1.00	3.19	0.39	1.49	0.05	4.08	0.43	1.72	0.00
*TOB1*	1.02	0.40	1.23	0.69	1.40	0.02	1.38	0.00	1.40	0.03	1.47	0.00
*ANKRA2*	1.07	0.44	1.39	0.27	1.63	0.01	1.48	0.02	1.60	0.02	1.60	0.00
*CD70*	2.45	0.55	2.04	0.75	4.44	0.04	2.50	0.04	5.05	0.03	3.01	0.00
*EDA2R*	8.96	0.61	1.31	1.00	19.58	0.87	1.57	0.02	31.64	0.29	1.67	0.00
*IGFBP4*	1.19	0.68	1.19	1.00	1.61	0.06	1.45	0.01	1.63	0.05	1.52	0.00

A comparison of the gene expression fold change (FC) responses obtained using microarray technology (MA) and qPCR at the three doses (0.5-1.5 Gy) of α-particle radiation examined. Transcripts that were shown to be differentially expressed at all three doses and the medium (1.0 Gy) and high (1.5 Gy) dose were validated using qPCR. PV indicates the p-value obtained using either ANOVA or microarray statistics. The table is divided into two sections; the first section is comprised of genes that were shown to be significant using MA technology at all three doses and the second section are those genes that were shown to be significant using MA technology at the medium (1.0 Gy) and high (1.5 Gy) dose.

### Custom qPCR panel

A customized gene array panel was developed to more cost-effectively assess whether the differentially responding genes observed from the microarray dataset were expressed in the WBC population, remained differentially expressed following exposure to α-particle radiation and were responsive to X-ray exposures.

**Figure 5:**
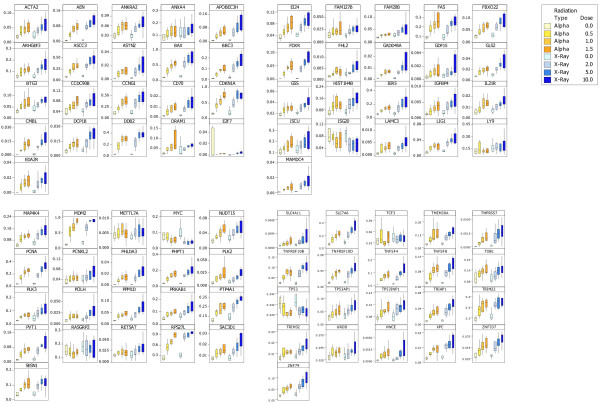
**Box-plot representation of the 84 gen custom array panel derived from the qPCR data of isolated white blood cells exposed to α-particle and X-ray radiation.** Genes are listed alphabetically and are plotted with 2^-ΔCT^values along the Y-axis. The central line represents the median of the data and the box edges represent the upper (75th) and lower (25th) percentile. Whiskers denote the highest and lowest values from the data set within the upper and lower limits. Limits are defined as 1.5*50 percentile spread.

**Figure 6:**
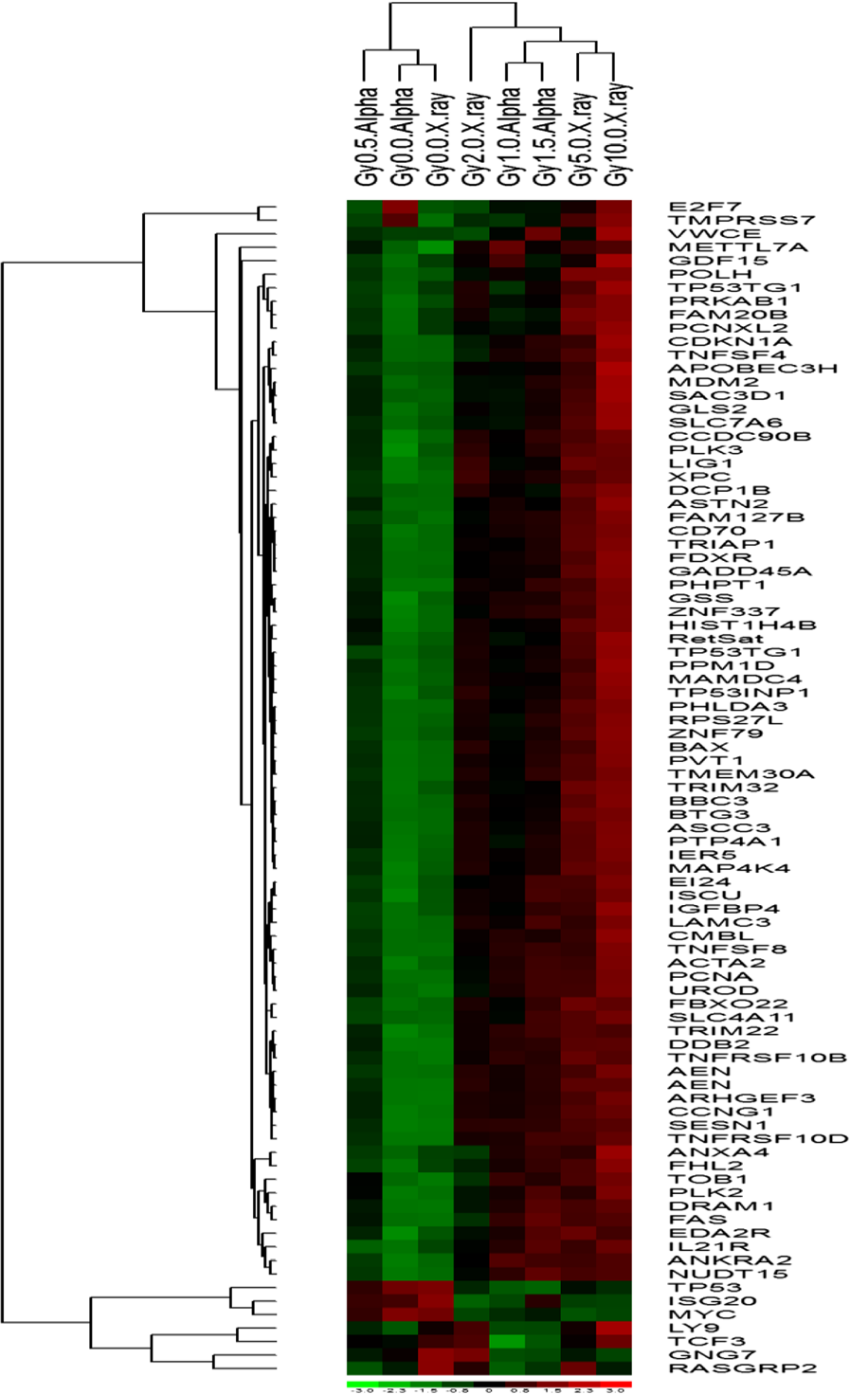
**Median based hierarchical clustering dataset to determine common groupings of samples and genes.** Dataset is obtained from qPCR results in isolated white blood cells exposed to α-particle and X-ray radiation.

WBCs were isolated from 12 healthy individuals and exposed to α-particles and X-rays. Total white blood cell counts were typically in the range of 5-10 × 10^6^ cells/mL. The viability of the cells was assessed using the Trypan Blue viability assay pre- and post-irradiation. The cells remained viable (above 98%) and no significant changes in blood cell counts or populations subsets were observed post-irradiation relative to unirradiated cells (Table 4). Twenty-four hours after irradiation, RNA was extracted and reverse transcribed to cDNA. A comparison of the differential gene responses obtained from the qPCR of WBCs and microarray analysis of PBMCs showed similar fold change and statistical significance for the majority of the 68 transcripts that were assessed (Table 5). These responding genes were compared to those obtained for X-ray exposed cells. Box-plots of the responding genes from the WBC qPCR dataset allowed for a visual comparison of the two radiation types and the range in inter-individual variability between transcripts (Figure 5). Overall, all genes responsive in α-particle treated cells were also observed to be expressed in X-irradiated cells. The data displayed minimal variability between control treatment groups under varied radiation exposure conditions. Furthermore, the majority of genes displayed dose-response trends for both α-particle and X-ray radiation. Hierarchical clustering (Figure 6) was further used to display groupings and make class distinctions. The two control groups clustered together as expected and showed a distinct trend relative to the other exposed groups. The lowest dose of radiation (0.5 Gy α-particle) also clustered with the unexposed groups. The next largest classification comprised the remaining exposure groups, in which the 2 Gy X-ray, 1.0 and 1.5 Gy α-particle and 5 and 10 Gy X-ray were classified further from the controls in order of descending similarity respectively. The subsequent clustering of the 1.0 and 1.5 Gy α-particle exposure and the 5 and 10 Gy X-ray exposure together suggests that it is possible to make distinctions between high X-ray radiation doses and α-particle doses using a clustering algorithm.

**Table 4:** A typical representation of complete blood counts from isolated white blood cells obtained from healthy individuals post-exposure

**Exposure**	**Viability**	**WBC total**	**Neutrophils**	**Lymphocytes**	**Monocytes**	**Eosinophil**	**Basophils**
**Condition (Gy)**	**(%)**	**(Cell/ml × 10**^**6**^**)**					
Alpha 0.0	98.0	4.0	1.78 (45%)	1.86 (47.1%)	0.26 (6.7%)	0.04 (1.1%)	0.00 (0.1%)
Alpha 0.5	99.0	3.8	1.69 (44.4%)	1.86 (48.9%)	0.22 (5.9%)	0.02 (0.6%)	0.01 (0.2%)
Alpha 1.0	99.0	3.8	1.73 (45.1%	1.86 (48.4%)	0.2 (5.3%)	0.04 (1.0%)	0.01 (0.2%)
Alpha 1.5	99.0	3.7	1.69 (48%)	1.76 (48%)	0.19 (5.1%)	0.03 (0.8%)	0.00 (0.00%)
X-Ray 0.0	99.0	3.8	1.76 (45.8%)	1.88 (48.9%)	0.17 (4.4%)	0.03 (0.8%)	0.00 (0.1%)
X-Ray 2.0	99.0	3.8	1.74 (46.3%)	1.82 (48.4%)	0.16 (4.2%)	0.03 (0.9%)	0.01 (0.2%)
X-Ray 5.0	99.0	3.7	1.70 (46%)	1.80 (48.9%)	0.13 (3.6%)	0.05 (1.4%)	0.00 (0.1%)
X-Ray 10.0	99.0	4.0	1.85 (45.7%)	2.03 (50.4%)	0.11 (2.8%)	0.04 (1.0%)	0.00 (0.1%)

**Table 5:** **WBC transcriptional profiling post *α*-particle and X-ray radiation via custom qPCR array**

**Radiation type**	**Alpha**						**X-ray**					
**Dose (Gy)**	**0.5**		**1.0**		**1.5**		**2.0**		**5.0**		**10.0**	
**Gene ID**	**FC**	**PV**	**FC**	**PV**	**FC**	**PV**	**FC**	**PV**	**FC**	**PV**	**FC**	**PV**
**All doses**												
*DDB2*	4.65	0.00	8.45	0.00	8.76	0.00	5.37	0.00	8.59	0.00	9.12	0.00
*PCNA*	3.30	0.00	6.05	0.00	7.27	0.00	3.80	0.00	5.76	0.00	7.47	0.00
*AEN*	6.02	0.01	12.22	0.00	13.86	0.00	12.67	0.00	15.88	0.00	18.58	0.00
*TNFSF4*	4.71	0.03	9.04	0.00	11.01	0.00	5.56	0.00	10.64	0.00	14.46	0.00
*PHPT1*	2.65	0.03	4.22	0.00	4.96	0.00	3.68	0.00	4.73	0.00	5.70	0.00
*TNFRSF10B*	2.29	0.03	3.94	0.00	3.52	0.00	3.66	0.00	5.27	0.00	5.47	0.00
*MAP4K4*	1.90	0.05	2.29	0.00	2.49	0.00	2.03	0.00	2.28	0.00	2.50	0.00
*GLS2*	2.00	0.06	2.45	0.00	2.62	0.00	2.66	0.00	3.73	0.00	4.37	0.00
*ACTA2*	1.99	0.07	2.48	0.00	2.90	0.00	1.95	0.01	2.35	0.00	2.82	0.00
*TRIAP1*	2.88	0.07	4.27	0.00	5.18	0.00	2.87	0.00	4.11	0.00	5.03	0.00
*IER5*	2.02	0.09	2.82	0.00	2.79	0.00	2.75	0.00	3.85	0.00	4.47	0.00
*APOBEC3H*	7.03	0.10	13.35	0.00	16.63	0.00	7.82	0.00	12.59	0.00	17.99	0.00
*TNFRSF10D*	2.64	0.10	3.83	0.00	4.91	0.00	3.49	0.00	3.81	0.00	4.66	0.00
*XPC*	2.43	0.13	4.00	0.00	2.99	0.00	3.76	0.00	3.89	0.00	4.28	0.00
*PPM1D*	2.21	0.13	2.65	0.02	3.18	0.00	2.17	0.00	2.81	0.00	3.55	0.00
*BAX*	3.35	0.15	5.35	0.00	6.56	0.00	5.21	0.00	5.83	0.00	7.19	0.00
*PHLDA3*	19.38	0.23	50.31	0.00	46.15	0.00	26.63	0.00	37.99	0.00	47.11	0.00
*ASTN2*	5.85	0.24	10.73	0.01	10.09	0.01	10.41	0.00	15.76	0.00	16.68	0.00
*GADD45A*	4.76	0.30	8.33	0.01	8.07	0.00	7.35	0.00	10.87	0.00	14.02	0.00
*MAMDC4*	3.32	0.36	4.77	0.03	4.98	0.01	3.86	0.00	5.09	0.00	5.68	0.00
*CMBL*	7.34	0.43	16.13	0.01	19.65	0.00	10.40	0.00	13.33	0.00	18.53	0.00
*SESN1*	1.83	0.55	2.86	0.04	3.45	0.00	2.41	0.02	2.72	0.04	2.54	0.02
*ASCC3*	2.82	0.53	4.35	0.08	5.48	0.01	4.06	0.00	4.63	0.02	5.45	0.00
*CCNG1*	2.12	0.55	3.35	0.05	4.22	0.00	2.88	0.02	3.23	0.03	3.80	0.00
*FBXO22*	1.70	0.66	2.41	0.16	3.10	0.02	2.39	0.01	2.63	0.06	2.93	0.01
*TP53INP1*	1.46	0.66	1.80	0.28	2.20	0.04	1.73	0.05	1.74	0.33	2.10	0.06
*FAS*	1.72	0.72	2.28	0.30	3.19	0.02	1.80	0.07	2.07	0.46	2.41	0.20
*RPS27L*	2.34	0.99	3.76	0.89	5.96	0.61	3.83	0.05	3.23	0.90	4.57	0.67
*TMEM30A*	1.91	0.99	2.82	0.87	4.04	0.56	2.82	0.06	2.70	0.90	3.42	0.67
**Med/High dose**												
*BBC3*	5.15	0.03	9.60	0.00	10.83	0.00	6.80	0.00	9.67	0.00	11.92	0.00
*TNFSF8*	2.98	0.03	5.64	0.00	6.38	0.00	4.57	0.00	5.65	0.00	7.34	0.00
*PVT1*	3.20	0.04	5.09	0.00	5.76	0.00	3.76	0.00	5.58	0.00	6.89	0.00
*FDXR*	10.88	0.05	24.04	0.00	27.24	0.00	17.01	0.00	27.84	0.00	37.91	0.00
*TRIM32*	1.80	0.05	1.96	0.01	2.01	0.01	1.71	0.01	2.11	0.00	2.48	0.00
*ANKRA2*	1.54	0.05	2.07	0.00	2.15	0.00	1.73	0.00	2.01	0.00	2.12	0.00
*GSS*	1.44	0.05	1.67	0.00	1.69	0.00	1.44	0.01	1.72	0.00	2.01	0.00
*HIST1H4B*	1.79	0.07	2.06	0.00	1.96	0.01	1.67	0.04	2.09	0.00	2.59	0.00
*ARHGEF3*	1.62	0.07	2.03	0.00	2.20	0.00	1.98	0.00	2.47	0.00	2.83	0.00
*SLC7A6*	2.37	0.09	3.19	0.00	3.94	0.00	2.42	0.00	3.25	0.00	4.28	0.00
*CD70*	5.76	0.13	10.08	0.00	12.92	0.00	6.14	0.00	9.06	0.00	9.89	0.00
*ZNF79*	1.96	0.13	2.58	0.00	3.17	0.00	2.18	0.00	2.95	0.00	3.68	0.00
*TOB1*	1.39	0.13	1.61	0.00	1.74	0.00	1.26	0.13	1.63	0.00	1.78	0.00
*ZNF337*	1.42	0.37	1.88	0.00	2.08	0.00	1.66	0.01	1.92	0.00	2.30	0.00
*PRKAB1*	1.67	0.38	2.06	0.05	2.32	0.02	1.81	0.01	2.06	0.01	2.38	0.00
*BTG3*	1.58	0.38	1.87	0.04	2.15	0.01	1.66	0.02	1.94	0.01	2.06	0.00
*POLH*	1.74	0.42	2.34	0.07	2.16	0.13	2.01	0.02	2.44	0.02	2.92	0.00
*MDM2*	3.83	0.43	6.10	0.05	8.91	0.00	3.76	0.01	5.24	0.01	8.33	0.00
*DCP1B*	1.84	0.46	2.59	0.04	2.37	0.07	2.47	0.00	2.98	0.00	3.39	0.00
*FAM20B*	1.41	0.53	1.54	0.23	1.60	0.17	1.50	0.02	1.73	0.01	1.91	0.00
*PCNXL2*	1.26	0.55	1.44	0.09	1.45	0.11	1.23	0.21	1.51	0.02	1.76	0.00
*ISG20*	-1.18	0.58	-1.53	0.11	-1.36	0.28	-1.55	0.49	-1.67	0.11	-1.61	0.18
*CDKN1A*	3.12	0.62	4.70	0.20	6.97	0.02	3.67	0.01	4.52	0.13	6.84	0.00
*LAMC3*	3.76	0.66	4.94	0.47	6.68	0.21	4.42	0.02	4.42	0.33	5.89	0.12
*DRAM1*	1.54	0.66	2.00	0.19	2.62	0.02	1.56	0.12	1.92	0.25	2.17	0.08
*RETSAT*	1.27	0.66	1.42	0.28	1.19	0.35	1.82	0.02	2.09	0.01	2.35	0.00
*ISCU*	1.25	0.66	1.43	0.22	1.86	0.01	1.45	0.03	1.53	0.11	1.62	0.02
*EDA2R*	13.97	0.73	30.23	0.36	39.14	0.15	11.63	0.02	14.28	0.27	13.00	0.20
*NUDT15*	1.48	0.74	1.85	0.23	2.66	0.03	1.73	0.03	1.84	0.25	1.91	0.14
*FHL2*	1.66	0.74	2.77	0.16	2.56	0.20	1.69	0.05	2.23	0.13	2.84	0.01
*CCDC90B*	1.42	0.88	1.68	0.53	2.28	0.04	1.56	0.22	1.57	0.83	1.82	0.42
*IGFBP4*	1.51	0.92	2.54	0.55	2.81	0.40	2.45	0.02	2.77	0.37	3.23	0.15
*SAC3D1*	1.46	0.94	1.69	0.77	2.01	0.56	2.04	0.04	2.12	0.51	2.85	0.17
*E2F7*	-1.98	0.94	-1.26	0.87	1.26	0.92	-1.00	0.31	1.56	0.51	2.61	0.50
*FAM127B*	1.57	0.98	1.56	0.90	2.18	0.52	1.94	0.06	1.74	0.90	1.96	0.76
*TMPRSS7*	1.66	0.99	1.55	0.73	2.56	0.76	4.16	0.13	5.92	0.34	8.32	0.05
*GDF15*	2.98	0.99	5.21	0.87	4.54	0.78	2.90	0.05	2.37	0.94	4.61	0.62
*TRIM22*	1.67	0.99	2.27	0.89	3.15	0.56	2.20	0.06	1.91	0.96	1.89	0.85
*TP53AP1*	1.37	0.99	1.62	0.90	2.32	0.49	1.73	0.07	1.53	0.97	1.95	0.77

### MicroRNA expression

The screening of ~800 miRNA transcripts using NanoString profiling resulted in a minimal number of responding targets. Only one miRNA (miR-34a) was observed to be differentially modulated (p < 0.05) across all doses following α-particle radiation exposure in WBC. This target was dose-responsive and subsequently validated using qPCR (Table 6). miR-34a was up-regulated over 2.5 fold in all exposed samples and had a similar 3 fold induction in the 1.0 and 1.5 Gy α-particle doses. This target was not specific to α-particle exposure as expression was also observed with X-ray irradiation at all three doses tested.

**Table 6:** qPCR validation of miRNA profiling results

**Radiation type**	**Alpha**						**X-ray**					
**Dose (Gy)**	**0.5**		**1.0**		**1.5**		**2.0**		**5.0**		**10.0**	
**miRNA**	**FC**	**PV**	**FC**	**PV**	**FC**	**PV**	**FC**	**PV**	**FC**	**PV**	**FC**	**PV**
*miR-34a*	2.67	0.00	3.05	0.00	3.14	0.00	3.14	0.00	3.30	0.00	3.05	0.00

Total isolated RNA from irradiated leukocytes was profiled with the nCounter system and subsequently validated via qPCR. Results are presented from n = 12 donors. FC = fold change, PV = p-value.

## Discussion

The overarching goal of this research was to identify genes responsive to α-particle radiation exposure for the purposes of developing effective triage tools for use in a population radiation exposure scenario. To date, the majority of genomic-based biomarker radiation studies have focused on photon radiation. Although there is a large body of work concerning radiation exposure and cytogenetic end-points, it was postulated that α-particle radiation may elicit differential cellular response due to its characteristic physical properties, which differ from photon radiation. This may potentially provide more accurate dose estimates for exposures and allow for differentiation between radiation types.

Circulating blood cells represent a sensitive target for early radiation damage and are easily accessible. Isolated leukocytes from healthy individuals were *ex-vivo* irradiated at a dose range of 0-1.5 Gy at 0.98 Gy/h. These doses were selected based on their relevance to an actual radiological dispersal device scenario [32], where the dose deposition is approximately 0.5 Gy per α-particle track [33]. Furthermore, previous studies from our laboratory have shown observable biological damage at this dose-range and a time-point of 24 hours post-exposure [34-36]. The X-ray exposure doses and dose rates were selected based on the work by Paul and Amundson [16]. Although whole blood was employed by Paul and Amundson, the limitations of our exposure system only allowed for the use of isolated leukocytes. It was observed that the red blood cells in whole blood minimized the transversal of α-particles through other cell types as measured by DNA double strand breaks via γ-H2AX (data not shown). Leukocytes exposed to α-particles displayed lower γ-H2AX intensities than the X-ray exposed samples possibly due to several factors. Firstly, the samples irradiated with X-rays were exposed to higher overall doses which increase the probability of inducing DNA double strand breaks. Secondly, the dose rates of exposure were markedly different. The α-particle exposure system is limited to a dose rate of ~1 Gy/hr and the X-ray exposure was performed at a dose rate of ~1 Gy/minute. This means the α-particle doses were delivered over a protracted amount of time relative to the X-rays. It has been previously documented that γ-H2AX foci reach a peak 30 minutes after exposure and then are resolved as repair is induced [37]. The time-scale of the higher doses (1.0, 1.5 Gy) of α-particle radiation exposure is between ~1-1.5 hours. Thus, there is a degree of repair occurring as the cells undergo irradiation and the measured signal is lowered due to resolved H2AX foci. There is an eventual equilibrium between induction and repair; it would be expected that the signal intensity would be higher for an acute exposure of an equivalent dose. All of the X-ray exposures were conducted on the order of minutes, with the highest (10 Gy) dose approximating 10 minutes.

A preliminary microarray screening following the exposure of isolated PBMC to α-particles identified 29 genes responsive at all doses tested (0.5-1.5 Gy) and 39 which responded only to the medium (1.0 Gy) and high (1.5 Gy) dose. These genes were validated using qPCR and shown through pathway analysis to be associated with signaling pathways centered around p53 and GADD45A, consistent with a DNA damage response (data not shown). To confirm the validity of this gene set in a more physiologically relevant population of cells, the complete white blood cell population was harvested. Furthermore, the ability of this gene panel to discriminate between radiation qualities was concurrently assessed using X-ray exposures. For this purpose, a customized gene panel was constructed using genes identified as significantly modulated by microarray analysis and included a selected few identified in the literature as being X-ray-responsive, primarily from the work conducted by Paul and Amundson [16].

The customized gene panel confirmed the validity of our initial microarray results. Primarily, genes differentially responsive in PBMC were also observed to be significantly expressed in the total WBC population using qPCR. However, a selected few genes were shown to lack statistical significance at 0.5 Gy, most likely due to the use of stringent statistical analysis accounting for multiple statistical comparisons and FDR correction using Benjamini-Hochberg testing [28]. When no FDR correction was employed, the qPCR dataset were more comparable to the PBMC microarray results. This is not unexpected as in comparison to microarrays, qPCR may report different statistical assignments [31,38].

Further visualization of the data using box plots allowed for an assessment of the gene responses with respect to individual variability. All control treatment groups displayed low inter-individual variability for the majority of differentially expressed transcripts, particularly between radiation types, highlighting the potential for these transcripts to be strong biomarkers. Furthermore, the majority of genes displayed dose-response trends for both α-particle and X-ray radiation. This is further highlighted in the hierarchical clustering of the dataset. Inputting all qPCR data resulted in the classification of treatment groups based on exposure doses. Clustering of the 1.0 and 1.5 Gy α-particle exposures together and the 5 and 10 Gy X-ray exposure suggests potential for distinctions to be made between high X-ray radiation doses and comparably lower α-particle doses.

Overall, the gene-expression changes induced by α-particle radiation were not distinct from the X-ray responses. Although all genes modulated in the α-particle exposed WBCs were also observed after X-ray exposure, there were differing degrees of induction. In a selected few genes, α-particle doses of 1.0 Gy at a dose rate of ~1 Gy/hr were able to elicit the same fold induction as a 10 Gy X-ray dose at ~ 1 Gy/min. Thus, despite there being an order of magnitude difference in dose and a greater than fifty fold difference in dose rate between the radiation exposures, a similar cellular response was observed. It has been previously established that the lesions caused by α-particle tracks display different repair kinetics and fidelity [39]. Only a 24 hour time point was examined in this study and there may be pronounced temporal differences in gene expression resulting from the differing degrees of damage and repair between the radiation types.

To date, Turtoi et al., [40] is the only other group to examine α-particle radiation induced genomic-wide transcriptional effects in isolated blood cells. This group employed a harvest time of 1hr post-irradiation using a dose range of 0.05 - 1.6 Gy of α-particle radiation. Three hundred and thirty nine genes were shown to be differentially modified with 54% up-regulated and 46% down-regulated. In comparison, the present study identified fewer genes, the majority of which were upregulated. These differences may be attributed to experimental conditions, as Turtoi et al., used varying dose-rates and a post-irradiation harvest time of 1 hour. As well, their gene responses were obtained from only one individual, whereas the present study used 12 different donors.

## Conclusion

In summary, genomic strategies were employed for the identification of gene-based responses in PBMCS and WBCs exposed to α-particle radiation. Genomic screening of PBMCs exposed to α-particle radiation identified twenty-nine transcripts that responded at 0.5, 1.0 and 1.5 Gy and thirty-nine genes were shown to be differentially modulated at exposures of 1.0 and 1.5 Gy. Subsequent comparison using WBCs with high dose-rate X-ray radiation showed that both radiation types elicited similar gene responses with varying degree of fold induction. No α-particle exclusive gene modulations were identified. Therefore, current gene panels for photon radiation may also be applicable for use in α-particle radiation biodosimetry. Future work includes testing the gene panel in an *in vivo* environment, using radiotherapy patients undergoing either total body irradiations or α-particle radiation therapy.

## Abbreviations

^241^Am: Americium; ^222^Rn: Radon; ^226^Ra: Radium; ^210^Po: Polonium; FBS: Fetal bovine serum; TBS: Triphosphate buffered saline; TST: TBS serum triton; PBS: Phosphate buffered saline; α: Alpha; MD: Mylar dish; RPMI: Royal Park Medical Institute; ANOVA: Analysis of variance; qRT-PCR: Quantitative real-time polymerase chain reaction; Ct: Comparative threshold; FC: Fold change; RDDs: Radiological dispersal devices; LET: Linear energy transfer; IPA: Ingenuity pathway analysis; CBC: Complete blood counts.

## Competing interest

The authors declare they have no competing interests.

## Authors’ contribution

VC contributed to the conception and design of the study, acquisition of data and analysis and interpretation of data and revising of manuscript. VC drafted the manuscript and provided final approval for publication. MH contributed to the execution of experiments, data analysis and interpretation and revising of manuscript. RW contributed to H2AX data analysis and final manuscript revision. All authors read and approved the final manuscript.

## Pre-publication history

The pre-publication history for this paper can be accessed here:

http://www.biomedcentral.com/1755-8794/7/43/prepub
